# EEG pre-burst suppression: characterization and inverse association with preoperative cognitive function in older adults

**DOI:** 10.3389/fnagi.2023.1229081

**Published:** 2023-08-30

**Authors:** Melody Reese, Soren Christensen, Harel Anolick, Kenneth C. Roberts, Megan K. Wong, Mary Cooter Wright, Leah Acker, Jeffrey N. Browndyke, Marty G. Woldorff, Miles Berger, the MADCO-PC and INTUIT Investigators, O Akinyemi

**Affiliations:** ^1^Department of Anesthesiology, School of Medicine, Duke University, Durham, NC, United States; ^2^Center for the Study of Aging and Human Development, Duke University Medical Center, Durham, NC, United States; ^3^Trinity College, Duke University, Durham, NC, United States; ^4^Pratt School of Engineering, Duke University, Durham, NC, United States; ^5^Center for Cognitive Neuroscience, Duke University, Durham, NC, United States; ^6^School of Medicine, Duke University, Durham, NC, United States; ^7^Department of Psychiatry, Duke University, Durham, NC, United States; ^8^Department of Psychology and Neuroscience, Duke University, Durham, NC, United States; ^9^Alzheimer’s Disease Research Center, Duke University, Durham, NC, United States

**Keywords:** burst suppression, perioperative, anesthesia, non-cardiac surgery, EEG, postoperative delirium, preoperative cognition, pre-burst suppression

## Abstract

The most common complication in older surgical patients is postoperative delirium (POD). POD is associated with preoperative cognitive impairment and longer durations of intraoperative burst suppression (BSup) – electroencephalography (EEG) with repeated periods of suppression (very low-voltage brain activity). However, BSup has modest sensitivity for predicting POD. We hypothesized that a brain state of lowered EEG power immediately precedes BSup, which we have termed “pre-burst suppression” (preBSup). Further, we hypothesized that even patients without BSup experience these preBSup transient reductions in EEG power, and that preBSup (like BSup) would be associated with preoperative cognitive function and delirium risk. Data included 83 32-channel intraoperative EEG recordings of the first hour of surgery from 2 prospective cohort studies of patients ≥age 60 scheduled for ≥2-h non-cardiac, non-neurologic surgery under general anesthesia (maintained with a potent inhaled anesthetic or a propofol infusion). Among patients with BSup, we defined preBSup as the difference in 3–35 Hz power (dB) during the 1-s preceding BSup relative to the average 3–35 Hz power of their intraoperative EEG recording. We then recorded the percentage of time that each patient spent in preBSup, including those without BSup. Next, we characterized the association between percentage of time in preBSup and (1) percentage of time in BSup, (2) preoperative cognitive function, and (3) POD incidence. The percentage of time in preBSup and BSup were correlated (Spearman’s ρ [95% CI]: 0.52 [0.34, 0.66], *p* < 0.001). The percentage of time in BSup, preBSup, or their combination were each inversely associated with preoperative cognitive function (β [95% CI]: −0.10 [−0.19, −0.01], *p* = 0.024; −0.04 [−0.06, −0.01], *p* = 0.009; −0.04 [−0.06, −0.01], *p* = 0.003, respectively). Consistent with prior literature, BSup was significantly associated with POD (odds ratio [95% CI]: 1.34 [1.01, 1.78], *p* = 0.043), though this association did not hold for preBSup (odds ratio [95% CI]: 1.04 [0.95, 1.14], *p* = 0.421). While all patients had ≥1 preBSup instance, only 20.5% of patients had ≥1 BSup instance. These exploratory findings suggest that future studies are warranted to further study the extent to which preBSup, even in the absence of BSup, can identify patients with impaired preoperative cognition and/or POD risk.

## 1. Introduction

As the number of global surgeries continues to increase beyond 300 million per year (Weiser et al., 2015), the number of surgical patients at risk for postoperative delirium will continue to rise. Postoperative delirium is a transient disturbance of mental status and attention following surgery, and is associated with extended hospital stays, increased dementia risk, and increased postoperative mortality (Deiner and Silverstein, 2009; Rengel et al., 2018). Postoperative delirium occurs at increased rates among older surgical patients, with an incidence of 12–53% in non-cardiac surgical patients over age 65 (Reddy et al., 2017). As the population ages (Jordan, 2020) and increased numbers of older adults undergo surgery (Daiello et al., 2019), understanding the etiology of postoperative delirium is a key research question in geriatric perioperative medicine.

Postoperative delirium has been associated with intraoperative burst suppression (BSup), periods of electroencephalogram (EEG) recordings in which quick bursts alternate with suppressed activity (Bennett et al., 2009; Shanker et al., 2021), a pattern that is thought to reflect decreased neuronal activity due to neuronal pathology, high anesthetic dosage, or hypothermia (Brown et al., 2010). BSup has also been associated with postoperative mortality (Willingham et al., 2014) and worse neurologic outcomes (Wennervirta et al., 2009), and some data suggests that patients who demonstrate BSup are at greater risk for developing postoperative delirium than those without BSup, though causal relations between BSup and postoperative delirium have been a subject of controversy (Soehle et al., 2015; Fritz et al., 2016; Wildes et al., 2019; Evered et al., 2021; Berger et al., 2023). Thus, the extent to which BSup actually contributes to cognitive impairment vs. the extent to which it is merely a marker of latent underlying neuropathology is unclear.

Despite its association with postoperative delirium, the utility of BSup as a clinical predictor is limited by the rarity of BSup in certain patient populations. Studies have reported a BSup incidence as low as 9% among surgical patients receiving general anesthesia with propofol and remifentanil (Besch et al., 2011). Further, variation in the frequency of BSup may be driven by differences in the method of BSup measurement (Muhlhofer et al., 2017), differences in patient characteristics such as age or surgery type, or variability in the use of EEG-guided anesthetic titration among surgical centers, along with other patient or surgical factors (Whitlock et al., 2014; Wildes et al., 2019). The rarity of BSup suggests it is likely to have low sensitivity as a predictor of postoperative delirium.

Although BSup may have low sensitivity for predicting postoperative delirium, in our clinical experience, a brief period of intermediate EEG suppression often precedes BSup epochs. Moreover, we have observed similar brief periods of intermediate EEG suppression even in patients who never had actual BSup. Thus, we hypothesized that an intermediate EEG suppression pattern, which we have termed pre-burst suppression (preBSup), tends to immediately precede BSup. We also hypothesized that the brain does not instantaneously switch into and out of BSup, but instead goes through a gradual and identifiable decline in EEG power (i.e., preBSup) just prior to BSup. Finally, we hypothesized that preBSup can occur on its own (i.e., in the absence of BSup), and that preBSup (like BSup) is associated with postoperative delirium. Thus, if patients spend more time in the intermediate state of preBSup than in full BSup, then preBSup could be a more sensitive predictor of postoperative delirium than BSup itself, including among patients who do not demonstrate actual BSup.

Aside from BSup, another risk factor for postoperative delirium is preoperative cognitive impairment. Preoperative cognitive function is assessed infrequently in routine clinical practice (Berger et al., 2018; Deiner et al., 2020; Peden et al., 2021), yet other delirium-associated intraoperative EEG patterns, such as low alpha power, have also been associated with both impaired preoperative cognition (Giattino et al., 2017) and postoperative delirium risk (Gutierrez et al., 2019). Thus, we also hypothesized that both intraoperative EEG preBSup and BSup would each be associated with impaired preoperative cognitive function. To investigate these hypotheses, we determined the extent to which a preBSup pattern is associated with BSup, and the extent to which preBsup, BSup, or their combination is associated with preoperative cognitive impairment and/or postoperative delirium incidence.

## 2. Materials and methods

### 2.1. Study population

**FIGURE 1:**
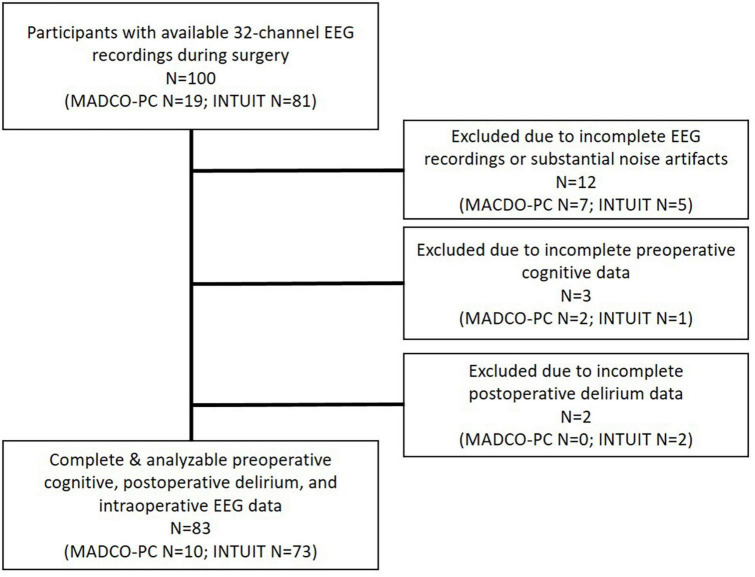
Consort diagram of participant data from the MADCO-PC and INTUIT studies.

In this study, we included all patients from 2 prior prospective observational cohort studies at Duke University Medical Center (Durham, NC) who underwent 32-channel intraoperative EEG recordings (Figure 1). These prior studies were Markers of Alzheimers Disease and Cognitive Outcomes After Perioperative Care (MADCO-PC; NCT01993836, 2013-2019) and Investigating Neuroinflammation Underlying Postoperative Cognitive Dysfunction (INTUIT; NCT03273335, 2017-2022) (Giattino et al., 2017; Berger et al., 2019). Both studies were registered with clinicaltrials.gov, and approved by the Duke Health Institutional Review Board. All study subjects or legally authorized representatives gave written informed consent before study participation. Both MADCO-PC and INTUIT enrolled Duke patients aged ≥60 years who were scheduled to undergo elective non-cardiac, non-neurologic surgery lasting ≥2 h with a planned postoperative overnight hospitalization. Exclusion criteria included incarceration and anticoagulant use that prohibited undergoing lumbar punctures. No exclusions were based on preoperative cognitive status; however, all enrolled participants were required to complete a cognitive test battery (described below) that required intact language function and adequate English fluency. Patient information such as demographics (age, sex, race), baseline clinical status, surgery type, and anesthesia type was obtained via surveys or chart review, as described (Berger et al., 2019). INTUIT study data were managed using REDCap electronic data capture at Duke University (Harris et al., 2009, 2019).

### 2.2. Cognitive testing and delirium assessment

To assess preoperative cognition, we used a well-established neurocognitive test battery (Newman et al., 2001; Mathew et al., 2013; Browndyke et al., 2017) that included the Randt Short Story Memory Test (Randt and Brown, 1983), the Modified Visual Reproduction Test from the Wechsler Memory Scale (Wechsler, 1981), the Digit Span Test from the revised version of the Wechsler Adult Intelligence Scale (WAIS-R) (Wechsler, 1981), the Digit Symbol Test from the WAIS-R (Wechsler, 1981), the Trail Making Test Part B (Reitan, 1958), and the Hopkins Verbal Learning Test (Brandt, 1991). Scores from these tests were then combined via factor analysis with oblique rotation to obtain factor scores for five cognitive domains: Randt (narrative) verbal memory, Hopkins (episodic) verbal memory, executive function, visual memory, and attention/concentration (McDonagh et al., 2010). An overall cognitive index was then obtained by averaging the scores from these cognitive domain factors. Our group has used this cognitive assessment method for >20 years, both to reduce redundancy among tests and to minimize the need for multiple comparison corrections (Newman et al., 2001; McDonagh et al., 2010; Giattino et al., 2017).

Delirium incidence was measured using the 3-Minute Confusion Assessment Method (3D-CAM) (Marcantonio et al., 2014) or the original Confusion Assessment Method (CAM) (Inouye et al., 1990). Participants were screened for delirium at baseline (before surgery) and twice daily after surgery for up to 5 days after surgery or until hospital discharge, whichever occurred first (Vasunilashorn et al., 2020).

### 2.3. Electroencephalogram recording

Due to funding limitations and/or COVID restrictions, 32-channel EEG recordings were performed on a consecutive set of 19 MADCO-PC patients, and on 81 INTUIT patients. A tethered EEG cap and recording system (BrainAmp MR Plus, Brain Products GmbH, Gilching, Germany) with a 32-channel custom electrode layout (Woldorff et al., 2002) were used for all MADCO-PC patients who underwent EEG recordings and for the initial 11 INTUIT study patients who underwent EEG recordings and were included in this study. To improve ease of use during surgery for subsequent INTUIT subjects who underwent EEG recordings, we switched to a wireless recording system (LiveAmp, Brain Products GmbH, Morrisville, NC, USA) using a 32-channel cap with standard international 10-10 EEG locations (Oostenveld and Praamstra, 2001).

Electrode impedances below 20 kΩ were obtained by light abrasion of the scalp locations with coarse electrode paste (Abralyte 2000, EASYCAP GmbH, Herrsching, Germany) before initiating data collection. EEG signals were recorded at a sampling rate ≥500 Hz with a 0.016–250 Hz band-pass filter. Procedure event markers, including time of induction, incision, and skin closure/end of surgery, were logged and cross-referenced with the surgical record to ensure accuracy.

### 2.4. Electroencephalogram preprocessing

Researchers blinded to patient cognitive and delirium status performed EEG processing in MATLAB (The MathWorks, Inc., Natick, MA, USA) using the EEGLAB toolbox (Delorme and Makeig, 2004) and custom scripts, as described (Giattino et al., 2017; Acker et al., 2021). We focused on EEG data from channel Fp1, given its clinical relevance; Fp1 is in the left medial frontal location where anesthesiologists typically place commercially available frontal EEG electrode strips to monitor brain responses to anesthesia.

Post-acquisition, the raw EEG data were band-pass filtered from 1–60 Hz to remove high-frequency noise, drift, and other artifacts. Epochs with false positives (marked “suppression” segments that were greater in amplitude than marked “burst” segments) and high amplitude artifacts (defined as >60 μV signals often reflecting large, high-frequency distortions from electrocautery or head movement) were removed (see Supplementary material for additional details). The data were downsampled to 250 Hz. Data from the standard international 10-10 EEG cap were referenced to AFz at acquisition; data from the custom tethered caps were referenced to Cz at acquisition. Thus, for subjects recorded with the custom cap, the average signal of 2 custom electrode locations slightly anterior to the standard 10-10 locations for F1 and F2 was subtracted from Fp1 to get the closest possible approximation to a 10-10 AFz reference. For our primary analyses, we used all available intraoperative EEG data from 5 min after surgical incision until 1 h later or 5 min before extubation, whichever occurred first. This approach avoided potential interactions between surgical case length and recorded time in BSup.

### 2.5. Pre-burst suppression calculations

**FIGURE 2:**
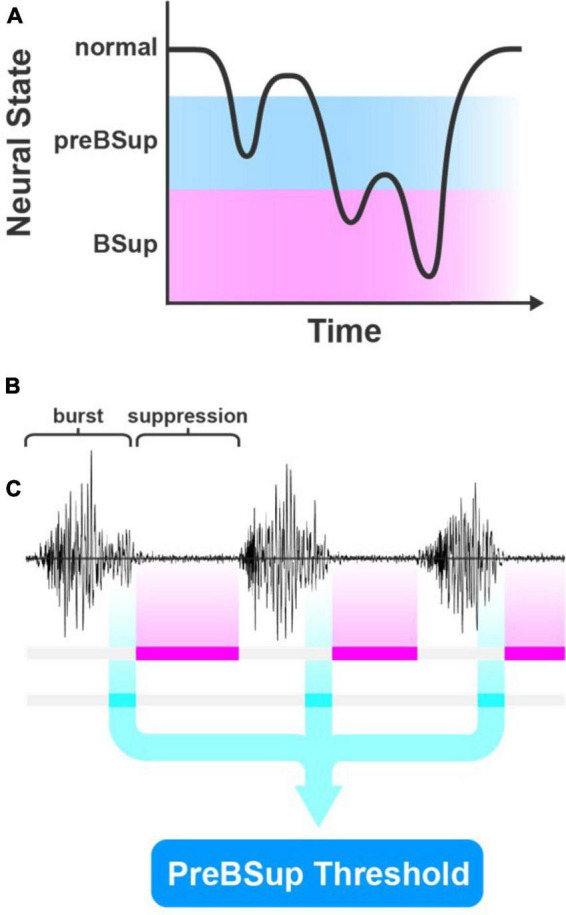
PreBSup concept and algorithm. **(A)** Conceptual model of pre-burst suppression (preBSup) as an intermediate neural state between the normal anesthetized state and burst suppression (BSup). **(B)** BSup is characterized by periods of repeated bursts of EEG activity separated by low-amplitude isoelectric activity called suppression. **(C)** In this study, instances of suppression were marked (magenta), and in subjects with >0 burst suppression instances, the 1 s of EEG data preceding each suppression instance (cyan) was extracted. These data were used to create a preBSup threshold, which was then used to mark preBSup in all subjects.

We hypothesized that a distinct EEG spectral power pattern would occur immediately before BSup in patients with BSup and that this pattern may also occur in patients without BSup (Figure 2A). Thus, we operationally defined preBSup as a total power-decrease threshold using data from subjects with >0 instances of BSup in their first hour of surgery. This threshold was then applied to the intraoperative EEG data of all subjects regardless of whether they had any BSup (Figures 2B, C).

**FIGURE 3:**
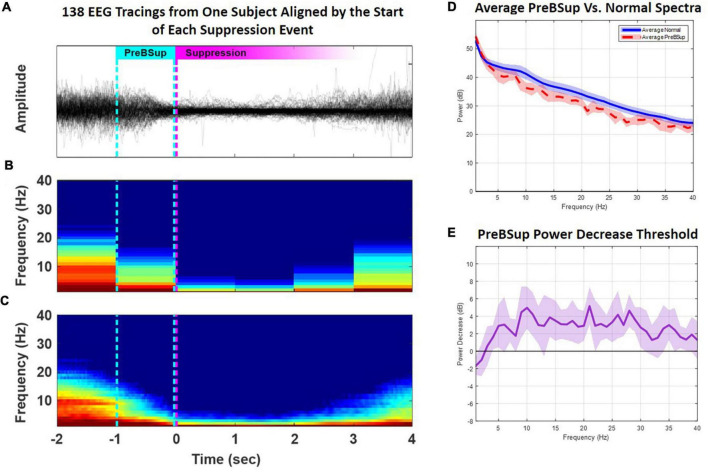
Creation of a preBSup threshold. In subjects with >0 BSup instances (*N* = 17), we created a preBSup threshold by calculating the average 3–35 Hz EEG power (dB) decrease for epochs occurring 1 s prior to suppression events relative to “normal spectra” during the rest of the EEG recording (excluding periods of BSup or artifact). **(A)** In an example subject, 138 instances of BSup from their surgical recording were overlaid and aligned to suppression onset (the magenta dotted line). The cyan dotted line represents the point in time 1 s before the onset of BSup in all 138 aligned EEG traces. **(B)** The average of all spectrograms from the 138 EEG traces plotted in panel **(A)** using non-overlapping 1-s windows. **(C)** The same data redrawn for smoother visualization using a 1-s moving window and a 0.025-s step size. **(D)** The averaged power across 1-s periods before suppression (i.e., average preBSup power, shown in red) among the 17 subjects with >0 BSup instances. The average normal spectra among the 17 subjects with >0 BSup instances are shown in blue. **(E)** The bold purple line indicates the average power (dB) decrease by frequency of the preBSup epoch [the red line in part **(D)**] from normal spectral power [the blue line in part **(D)**] with a 95% confidence interval depicted in lighter purple. We used the average power decrease from 3–35 Hz (a 2.32 dB drop) from the 17 subjects with >0 BSup instances as our threshold to detect and mark preBSup in all subjects, independent of BSup.

To define this power-decrease threshold, we first used a modified BSup algorithm to mark instances of BSup in every subject’s EEG recording (see Supplementary material) (Westover et al., 2013). Then, in each subject with >0 instances of intraoperative BSup (*N* = 17), we isolated the 1 s of EEG data preceding each suppression instance (i.e., preBSup). We chose a 1-s window for defining preBSup based upon visual inspection of the EEG power spectra just prior to BSup events. This 1-s window captured the decrease in power before suppression and excluded the preceding periods of average intraoperative power (Figure 3A). Next, we averaged the spectral power distribution of all these 1-s segments to create an average preBSup spectrum for that subject (Figures 3B, C). To determine the decrease in log-adjusted power (dB) associated with preBSup, we subtracted the aforementioned average preBSup spectrum from the spectral average of the subject’s entire recording, excluding epochs with BSup, preBSup, or artifacts (Figure 3D). This subtraction of dB power gave power-decrease-by-frequency information for that subject. Finally, we averaged these subject-specific power-decrease data across the 17 subjects with >0 instances of BSup, resulting in the final preBSup power-decrease threshold (Figure 3D, bold line). This final threshold was our working definition of preBSup, which we then used to mark preBSup epochs in all patients, regardless of whether they had any instances of BSup.

This preBSup definition specifies a relative decrease in power at each frequency rather than an absolute power decrease, which helps to account for variability in each subject’s baseline EEG power. Here, we took the average across-threshold values from 3–35 Hz in Figure 3D to form a generalized power decrease threshold for defining preBSup in all patients. Over the 3–35 Hz interval, the bold line indicates an average 2.32 dB drop in spectral power (equivalent to a 41% decrease in power on a linear scale) compared to that patient’s average 3–35 Hz intraoperative power spectrum. Thus, 1-s epochs were marked as preBSup if their spectral power in that second decreased from their average intraoperative spectrum power by at least 2.32 dB (the power decrease threshold). If preBsup overlapped with BSup in a given second, the time in preBSup was recorded as 1 s minus the duration of BSup in that second. After marking these instances of preBSup, we recorded the percentage of each patient’s case spent in preBSup and in BSup (beginning 5 min after surgical incision until 1 h later or 5 min prior to extubation, whichever occurred first).

### 2.6. Statistical analysis

All statistical analyses were performed by a statistician who was not involved in EEG data pre-processing. We summarized our patient cohort overall and by postoperative delirium status in Table 1. Spearman’s correlation tests were measured between the case percentage of preBSup and BSup, and Wilcoxon Rank Sums tests were used to assess differences in the case percentage of preBSup among patients who did vs. did not have BSup. Then, we evaluated the association between preBSup and both preoperative cognition and postoperative delirium incidence. For the association between preBSup and preoperative cognition, we examined the relationship between preBSup with both overall cognitive index and the 5 individual cognitive domains.

**TABLE 1:** Descriptive statistics of the study cohort.

Variable	Overall *N* = 83	No postoperative delirium *N* = 71	Postoperative delirium *N* = 12	*P*-value
Age	68 [64, 72]	67 [64, 72]	69.5 [64, 77.5]	0.340^1^
Male sex	43 (51.81%)	34 (47.89%)	9 (75.00%)	0.082^2^
White race	66 (79.52%)	60 (84.51%)	6 (50.00%)	0.014^3^
BMI	29.61 (5.51)	29.84 (5.44)	28.27 (5.98)	0.363^4^
APOE4 positive*	21 (25.93%)	19 (27.14%)	2 (18.18%)	0.719^3^
**ASA class**				
2	21 (25.30%)	19 (26.76%)	2 (16.67%)	0.563^3^
3	57 (68.67%)	47 (66.20%)	10 (83.33%)	
4	5 (6.02%)	5 (7.04%)	0 (0.00%)	
**Surgical service**				
General surgery	21 (25.30%)	19 (26.76%)	2 (16.67%)	0.063^3^
Orthopedics	13 (15.66%)	13 (18.31%)	0 (0.00%)	
Other	9 (10.84%)	9 (12.68%)	0 (0.00%)	
Thoracic	18 (21.69%)	15 (21.13%)	3 (25.00%)	
Urology	22 (26.51%)	15 (21.13%)	7 (58.33%)	
MMSE score < 25*	8 (9.88%)	4 (5.71%)	4 (36.36%)	0.010^3^
Years of education	16 [13, 17]	16 [14, 17]	13 [11.5, 16]	0.013^1^

Data are mean (SD), median [Q1, Q3], or *N* (%).

^1^Wilcoxon Rank Sums test.

^2^Chi-Square test.

^3^Fisher’s Exact test.

^4^*T*-test.

*2 of the 83 subjects had missing APOE4 data, MMSE data.

Associations between preoperative cognition and (1) BSup, (2) preBSup, and (3) the combination of BSup and preBSup were measured via linear regression analyses. The associations between BSup, preBSup, and their combination with postoperative delirium incidence were examined via Firth-corrected logistic regression models. Our independent variables for these models included case percentages of (1) PreBSup, (2) BSup, and (3) combined BSup and preBSup to evaluate potential differential effects of these EEG measures on preoperative cognition and postoperative delirium. Spearman’s correlations between preoperative cognition and intraoperative medication dosages or rates of administration were used to identify possible confounders to include in the general linear models. Given the low incidence of postoperative delirium in this cohort, we could only reasonably perform univariable analyses for delirium.

Due to the exploratory nature of this study, analyses were not corrected for multiple comparisons. Thus, findings were considered significant when *p* < 0.05. All statistical analyses were performed using SAS Studio 3.81 (SAS Institute, Cary, NC, USA).

## 3. Results

Preoperative characteristics for the 83 patients who had complete EEG and delirium data are presented in Table 1 (see Figure 1 for the participant flow diagram). Consistent with prior work (Guan et al., 2022), patients who later developed postoperative delirium had lower baseline MMSE scores and fewer years of education (Table 1). We first investigated the association between preBSup and BSup to determine whether these are related neurophysiologic patterns. The percentage of time spent in preBSup correlated with the percentage of time spent in BSup (Spearman’s ρ [95% CI]: 0.52 [0.34, 0.66], *p* < 0.001). Further, the percentage of time spent in preBSup differed significantly among patients with vs. without BSup (median [Q1, Q3]: 16.61 [13.73, 17.26] vs. 8.58 [6.44, 11.23] percentage of the first case hour, respectively; median of differences [95% CI]: 7.29 [5.26, 9.01], *p* < 0.001). While all patients had preBSup, only 17 patients (20.5%) had BSup.

**FIGURE 4:**
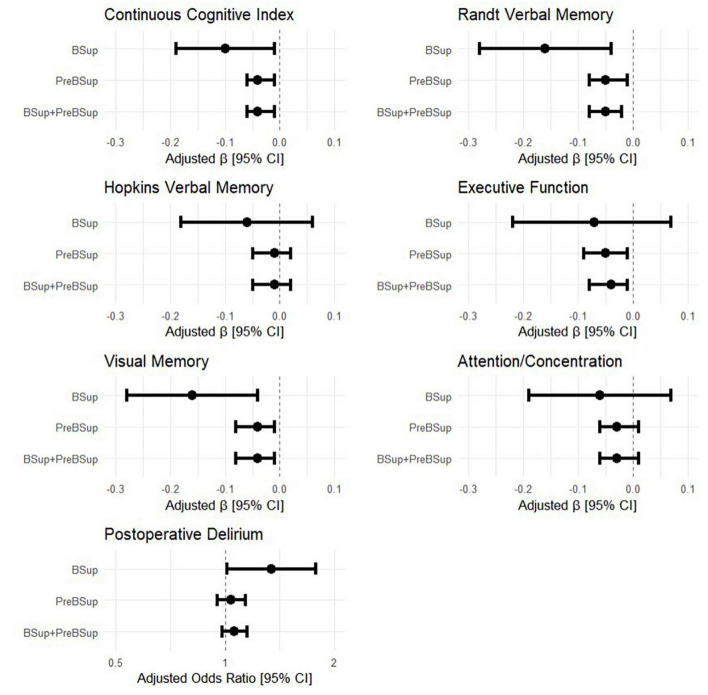
Forest plots of the relationship between intraoperative EEG patterns (percentage of first hour of surgical case spent in BSup, preBSup, or their combination) and preoperative cognitive measures and postoperative delirium incidence. Each point with error bars represents the linear regression beta coefficient and 95% confidence interval from a separate statistical model, for the effects of BSup, preBSup, or their combination on continuous cognitive index and the 5 preoperative cognitive factor domains (Randt verbal memory, Hopkins verbal memory, executive function, visual memory, and attention/concentration). The odds ratios from the simple Firth-corrected logistic regression models of the effect of BSup, preBSup, and their combination on postoperative delirium incidence are shown in the bottom panel.

Univariable models showed an association between overall preoperative cognitive function and percentages of time in (1) BSup (β [95% CI]: −0.10 [−0.19, −0.01], *p* = 0.024, R^2^ = 0.061), (2) preBSup β [95% CI]: (−0.04 [−0.06, −0.01], *p* = 0.009, R^2^ = 0.081), and (3) the combination of preBSup and BSup (β [95% CI]: −0.04 [−0.06, −0.01], *p* = 0.003, R^2^ = 0.101; Figure 4). Preoperative Randt verbal memory scores (the unstructured memory domain) were significantly associated with the percentage of time in all 3 intraoperative EEG states (BSup, preBSup, and combined preBSup and BSup). In contrast, preoperative Hopkins verbal memory scores (i.e., the structured verbal memory domain) were not associated with percentage of time in any of these EEG states (see Figure 4 and Table 2 for the β [95% CI] coefficients for each cognitive domain). Preoperative visual memory was associated with percentage of time in BSup, preBSup, and the combined percentage of time in BSup or preBSup, while executive function was only associated with preBSup and the combined preBSup and BSup percentages (Figure 4 and Table 2). Preoperative attention/concentration was not associated with any of these intraoperative EEG states (Figure 4 and Table 2).

**TABLE 2:** Univariable linear models of preoperative cognitive domains by intraoperative EEG percentages (model *N* = 83).

	Association strength between cognitive domain and% of time in each EEG state		
	Beta [95% CI]	*P*-value	R^2^
**Randt verbal memory**			
BSup model	−0.16 [−0.28, −0.04]	0.008	0.083
preBSup model	−0.05 [−0.08, −0.01]	0.010	0.078
Combined BSup and preBSup model	−0.05 [−0.08, −0.02]	0.003	0.106
**Hopkins verbal memory**			
BSup model	−0.06 [−0.18, 0.06]	0.330	0.012
preBSup model	−0.01 [−0.05, 0.02]	0.490	0.006
Combined BSup and preBSup model	−0.01 [−0.05, 0.02]	0.383	0.009
**Executive function**			
BSup model	−0.07 [−0.22, 0.07]	0.307	0.013
preBSup model	−0.05 [−0.09, −0.01]	0.021	0.064
Combined BSup and preBSup model	−0.04 [−0.08, −0.01]	0.020	0.065
**Visual memory**			
BSup model	−0.16 [−0.28, −0.04]	0.010	0.079
preBSup model	−0.04 [−0.08, −0.01]	0.023	0.062
Combined BSup and preBSup model	−0.04 [−0.08, −0.01]	0.007	0.088
**Attention/Concentration**			
BSup model	−0.06 [−0.19, 0.07]	0.325	0.012
preBSup model	−0.03 [−0.06, 0.01]	0.167	0.023
Combined BSup and preBSup model	−0.03 [−0.06, 0.01]	0.136	0.027

Cognitive domains are listed in bold in the left column, EEG models for BSup, preBSup, and the combination of both are listed under each cognitive domain.

Next, we examined the possibility that the relationship between EEG findings and preoperative cognition might be confounded by differential anesthetic dosage associated with preoperative cognition, i.e., if case anesthesiologists administered lower drug doses to patients who may have appeared to have pre-existing cognitive impairment. However, individual intraoperative medication administration rates and dosage(s) did not significantly differ in association with preoperative cognitive function (Supplementary Tables 1, 2). Thus, we found no evidence that intraoperative medications were potentially confounding the relationship between preoperative cognition and intraoperative EEG, since a confounder must be associated with both independent and dependent variables in an analysis. As such, we did not include intraoperative medication administration and dosage in our cognitive models.

Next, to determine whether preoperative cognition or intraoperative EEG parameters were associated with increased postoperative delirium risk, we analyzed univariable associations between postoperative delirium risk and both (1) preoperative cognition and (2) the percentage of time spent in preBSup, BSup, or their combination. Preoperative cognition was significantly associated with postoperative delirium incidence (odds ratio [95% CI]: 0.14 [0.05, 0.39], *p* < 0.001). The percentage of time patients spent in BSup was associated with postoperative delirium risk (odds ratio [95% CI]: 1.34 [1.01, 1.78], *p* = 0.043). However, no significant relationship was found between postoperative delirium and the percentage of time spent in preBSup (odds ratio [95% CI]: 1.04 [0.95, 1.14], *p* = 0.421) or the percentage of total time spent in either preSup or BSup (odds ratio [95% CI]: 1.06 [0.98, 1.15], *p* = 0.149. Further, we found no association between postoperative delirium and the rates of administration or dosage of intraoperative medications (Supplementary Table 3).

Among patients who developed postoperative delirium (*N* = 12), only 4 (33.3%) had BSup. Thus, while all patients (regardless of delirium status) experienced preBSup, we hypothesized that a set percentage of case time in preBSup may be a more sensitive measure than BSup for postoperative delirium. To investigate this potential sensitivity vs. specificity trade-off for BSup and preBSup in association with postoperative delirium, we used the Youden Index (J, where J = max [sensitivity + specificity – 1]) to generate optimal cut-points for percentage of time spent in BSup and preBsup among all patients. The Youden Index identifies the cut-point at which a biomarker (e.g., preBSup) is maximally effective for predicting an outcome (e.g., postoperative delirium) (Schisterman et al., 2008). Using a cut-point of ≥16.3% of time in preBsup had a sensitivity of 0.50 and a specificity of 0.75 (at *J* = 0.25) for postoperative delirium, whereas a cut-point of ≥1.34% of time in BSup had a sensitivity of 0.25 and specificity of 0.96 (at *J* = 0.21), confirming this trade-off in sensitivity versus specificity for these two measures.

## 4. Discussion

In this exploratory study, we defined and described an intraoperative neurophysiologic pattern that we termed pre-burst suppression (preBSup), which precedes EEG burst suppression (BSup) but can also occur in the absence of BSup. Since an extensive literature has shown an association between BSup and the risk for postoperative delirium, and between postoperative delirium and impaired preoperative cognition, we reasoned that BSup—and by extension, preBSup—would be associated with impaired preoperative cognition. We found that (1) preBSup was associated with BSup, and (2) preBSup, BSup, and the combination of both were each associated with preoperative cognition.

As expected, many more of our study subjects displayed preBSup than BSup; subjects who had no BSup still had measurable preBSup, as characterized by the preBSup pattern(s) extracted from subjects with BSup. Even in the subset of patients who had BSup, the total duration of preBSup was longer than the duration of BSup. Further, the average duration of preBSup was more variable than BSup duration. Thus, there may be greater statistical power for examining relationships between preBSup (rather than BSup) and both cognitive function and delirium risk.

Despite some previous studies with negative findings (Wildes et al., 2019), prior literature largely supports an association between BSup and postoperative delirium (Soehle et al., 2015; Fritz et al., 2016, 2018). Additionally, in a mediation analysis, Fritz et al. (2020) found that BSup mediates a small portion of the relationship between preoperative cognitive impairment and postoperative delirium. We have replicated the finding that BSup is associated with both postoperative delirium and impaired preoperative cognition, and the finding that preoperative cognition is associated with postoperative delirium. This fits with the notion that BSup may be associated with postoperative delirium not because it represents the brain’s response to excessive anesthetic doses, but rather because it represents the response of a vulnerable brain—already at risk for postoperative delirium—to normal anesthetic doses.

We further extended these findings by operationalizing preBSup, a distinct but related EEG pattern to BSup. Interestingly, preBSup was associated with preoperative executive function but not with postoperative delirium, while BSup was related to postoperative delirium but not with preoperative executive function. Yet both preBSup and BSup were associated with overall preoperative cognitive function (Figure 4). There are 3 major possible reasons for these relationships. (1) This pattern of results could represent a sensitivity vs. specificity trade-off between BSup and preBSup (i.e., preBSup could have increased ability to detect true positives for delirium over BSup but also an increased rate of false positives for delirium). Indeed, using the Youden Index, preBsup had greater sensitivity but less specificity than BSup in its association with postoperative delirium. Thus, while the true association between percentage of time spent in preBSup and postoperative delirium is unclear based on data from this cohort alone, it is possible that preBSup could serve as a more sensitive tool for perioperative monitoring and prevention of postoperative delirium, though future studies would be needed to identify the safety and efficacy of preBSup for this purpose. (2) The magnitude of effect between preBSup and postoperative delirium may be more subtle than that of BSup and postoperative delirium (with odds ratios of ∼1.15 vs. 1.34, respectively), and this study may simply not have been adequately powered to detect such a small association between preBSup and postoperative delirium. (3) Since these 2 different EEG patterns (preBSup and BSup) were associated with different cognitive phenotypes (postoperative delirium vs. preoperative executive function, respectively), different neural mechanisms may underlie BSup and preBSup, an important question for future study. More specifically, preBSup may be associated with preoperative cognitive impairments but possibly not the type, pattern, or degree that leads to postoperative delirium or later neurocognitive dysfunction, while BSup may reflect a type or severity of network dysfunction related more specifically to postoperative delirium. As an example, the change in neural activity reflected by the reduction in power in preBSup (which is not as extreme as the power reductions seen in the isoelectric suppressions of BSup) may not be sufficient to account for neural changes in postoperative delirium. Given the effect sizes seen here, a much larger study would be required to provide sufficient power to test the association between preBSup and postoperative delirium or to perform mediation analyses, as Fritz et al. (2020) did for the relationships between preoperative cognition, BSup, and postoperative delirium.

There are several limitations to this study. First, to reduce the chance of falsely labeling epochs as BSup, we utilized a smoothing protocol to produce continuous suppression markings (see Supplementary material for details on smoothing procedures.) However, while this technique can reduce the chance of falsely marking epochs as BSup, it does so at the cost of potentially missing short periods of real suppression.

Second, we did not correct for multiple comparisons here, since this was an initial exploratory study of preBSup. Thus, our results should be viewed as hypothesis-generating rather than hypothesis-proving or -confirming. Third, this was a single-center study that enrolled mostly Caucasian participants, which limits the potential generalizability of the study conclusions. Fourth, the sample size was relatively small in this study, particularly for subgroups such as patients with vs. without postoperative delirium. Low power for these subgroup analyses increases the risk for type II statistical errors, which highlights a need for larger studies to investigate whether there may be brain mechanism differences that underlie differential associations between preBSup and BSup, and preoperative cognition and postoperative delirium, respectively. Fifth, we defined preBSup based on 1-s epochs prior to BSup in the 17 patients with >0 BSup events. It is unclear how the definition of preBSup would change if it was based on a larger or different group of individuals with >0 BSup events, and whether any such changes in the preBSup definition would modulate its associations with delirium and preoperative cognitive function.

Finally, this is the first study to discuss the concept of preBSup and to define it using quantitative criteria. While we used the average 3–35 Hz power decrease 1 s prior to BSup, we recognize that there are many other ways in which to define the concept of preBSup. Alternatively, for example, preBSup could be defined by quantifying the median of the power decrease before BSup, the slope or shape of the power decrease before BSup, by analyzing frequency-specific changes in power (such as 8–12 Hz alpha) prior to BSup epochs, or via other methods. Ultimately, the concept of preBSup could be defined/operationalized in a number of different ways, and future studies will be needed to determine which definition of preBSup would provide the most useful information about the neurocognitive function of individual patients.

Additional studies will be required to determine the extent of neurophysiologic differences (e.g., in brain network activity or in connectivity patterns) between preBSup and BSup, yet the results presented here suggest that preBSup may add value beyond that of BSup for identifying patients with impaired preoperative cognition and/or postoperative delirium risk. There are other areas of research in which lower EEG amplitudes [e.g., discontinuity in infants (Yuan et al., 2023)] have been observed, and the extent to which these patterns are related to BSup and preBSup may be another important area for future study. Additional work would be needed to understand the underlying neurophysiological mechanisms of preBsup and the changes in neural activity (e.g., periodic vs. aperiodic) that occur before the onset of BSup itself, especially in patients who later develop perioperative neurocognitive disorders such as delirium.

## Data availability statement

The raw data supporting the conclusions of this article will be made available by the authors, without undue reservation.

## Ethics statement

The studies involving humans were approved by the Duke Health Institutional Review Board. The studies were conducted in accordance with the local legislation and institutional requirements. The participants provided their written informed consent to participate in this study.

## Author contributions

MR, SC, HA, KR, LA, MGW, and MB: study concept and design. MR, SC, HA, KR, MGW, and MB: EEG data processing and analysis. MR: statistical analysis. MR, SC, HA, KR, MCW, LA, MGW, and MB: data interpretation. MR, SC, HA, KR, MKW, MCW, LA, MGW, and MB: manuscript preparation. All authors contributed to the article and approved the submitted version.
