# Supplementary material for: EEG pre-burst suppression: characterization and inverse association with preoperative cognitive function in older adults
Source: Front Aging Neurosci. 2023 Aug 30;15:1229081. doi: 10.3389/fnagi.2023.1229081 (PMC10499509; doi:10.3389/fnagi.2023.1229081)
Supplement: Supplementary file 1 [file Data_Sheet_1.docx]

Supplementary Material

EEG Pre-Burst Suppression: Characterization and Inverse Association with Preoperative Cognitive Function in Older Adults

Melody Reese^†^; Soren Christensen^†^; Harel Anolick^†^; Kenneth Roberts; Megan K Wong; Mary Cooter Wright; Leah Acker; Jeffrey Browndyke; Marty Woldorff; Miles Berger; for the MADCO-PC and INTUIT Investigators

^†^These authors contributed equally to this work, and share first authorship.

*** Correspondence:** Dr Miles Berger: miles.berger@duke.edu

# Supplementary Data

## Burst Suppression Detection

A modified form of Westover’s variance-based burst suppression algorithm was used to detect instances of bust suppression.^1^ The original BSup algorithm thresholds a recursive estimate of local signal variance using a 3-step process at each EEG data point: 1) calculate a recency-focused mean, 2) calculate a recency-focused variation using this mean, and 3) compare this variation to a classification threshold θ. The recency-focused aspect of the first 2 steps uses the same forgetting factor, β, to steadily decrease the impact of all previous points on the current point’s binary classification as BSup. Importantly, in this algorithm, unlike a sliding window in which any given data point has no impact on exiting the trailing end of the window, all points before any given point have some impact on classification. The 3 steps are, as defined here, followed by the equation for forgetting factor β (τ is the forgetting time, the time required for 1 after a sequence of 0s, to decay to a value of 1/*e* ≈ 0.37):

$$\begin{matrix} {1) \mu}_{t}=\beta\mu_{t-1}+\left( 1-\beta\right)x_{t} \\ {2) \sigma}_{t}^{2}=\beta\sigma_{t-1}^{2}+\left( 1-\beta\right)\left( x_{t}-\mu_{t} \right)^{2} \\ {3) z}_{t}=\delta\left[ \sigma_{t}^{2}<\theta\right] \end{matrix}$$

$$\tau=-1/\left( F_{s}ln\beta\right)$$

As defined in Westover et al, *x_t_* is a single-channel EEG signal (amplitude in µV) at time *t,* *μ_t_* is the current value of the local signal mean, and $\sigma_{t}^{2}$ is the current value of the local signal variance. In 2), $\delta\left[ \sigma_{t}^{2}<\theta\right]=1$ when $\sigma_{t}^{2}<\theta$ and $\delta\left[ \sigma_{t}^{2}<\theta\right]=0$ when $\sigma_{t}^{2}\geq\theta$. Thus, *z_t_* becomes a binary sequence of zeros for points marked as bursts and ones for points marked as suppressions.

The optimal forgetting time τ was determined to be 104.7 ms (with ~95.5% accuracy) by Westover et al through optimization to match 20 manual expert EEG examinations of BSup data. Whereas the 200-Hz sampling rate used in Westover’s paper required a β of 0.9534, our downsampled sampling rate of 250 Hz required a β of 0.9625. An and colleagues set the classification threshold θ to 1.75 after visually scoring 6 candidate thresholds ranging from 1.4 to 3.5.^2^

The point-by-point thresholding method of this algorithm would often mark individual points as BSup in the middle of otherwise standard data, or else split BSup segments with a single-point non-BSup marking. Since the preBSup marking procedure requires knowing when each BSup instance begins, we added a 2-step smoothing protocol to this BSup algorithm: 1) We removed all BSup markings shorter than a certain length, and 2) we conjoined all remaining BSup markings closely enough to be considered one segment. The resulting smoothed BSup had clear start and end points to each suppression instance, and had significantly less noise that otherwise would have impeded the process of collecting preBSup data from the one-second preceding BSup onset. Burst patterns occurred on the order of seconds.

A second problem we encountered in using the Westover et al BSup algorithm was the tendency to mark false-positive instances of suppression in low-amplitude regions of a recording during which no BSup is present. To address this problem, we implemented a check that compares the amplitude of the suppression of each BSup instance to that of the preceding burst. Since bursts should (by definition of BSup) have a significantly greater amplitude than suppressions, we rejected all BSup instances in which the interquartile range (IQR) of the suppression amplitude was greater than 1/10^th^ of the maximum amplitude of the previous 10 seconds. The IQR was used for the suppression because the BSup algorithm sometimes lagged when terminating the suppression marking, thus ‘catching’ the high-amplitude at the beginning of the following burst. The maximum amplitude was used for the previous 10 seconds with the idea that if there was any overly noisy data preceding the suppression incident, the entire BSup instance would be compromised and thus the preceding one second of data would not reflect a true preBSup pattern. We manually chose the 1/10^th^ comparison factor by visual inspection.

1. Westover MB, Ching S, Shafi MM, Cash SS, Brown EN: Real-time segmentation and tracking of brain metabolic state in ICU EEG recordings of burst suppression. Annu Int Conf IEEE Eng Med Biol Soc 2013; 2013: 7108-11

2. An J, Jonnalagadda D, Moura V, Purdon PL, Brown EN, Westover MB: Spatial variation in automated burst suppression detection in pharmacologically induced coma. Annu Int Conf IEEE Eng Med Biol Soc 2015; 2015: 7430-3

# Supplementary Tables

**Supplemental Table 1.** Wilcoxon Rank Sum tests for preoperative cognitive index values among patients who did vs did not receive certain intraoperative medications.

| **Intraoperative Medication** | **Number of Patients Receiving the Drug (%)** | **Hodges-Lehmann Estimation (median of differences [95% CI])** | **P-Value** |
| --- | --- | --- | --- |
| Acetaminophen | 19 (22.9%) | 0.03 [-0.34, 0.41] | 0.884 |
| Succinylcholine | 12 (14.5%) | 0.09 [-0.38, 0.55] | 0.712 |
| Ketorolac | 10 (12.0%) | 0.25 [-0.24, 0.76] | 0.311 |
| Cisatracurium | 1 (1.20%) | 0.39 [-0.76, 2.85] | 0.392 |
| Ketamine | 24 (28.9%) | -0.09 [-0.40, 0.25] | 0.641 |
| Midazolam | 14 (16.9%) | 0.20 [-0.23, 0.55] | 0.334 |
| Dexmedetomidine | 18 (21.7%) | 0.17 [-0.19, 0.55] | 0.291 |
| RE Paralytics | 68 (81.9%) | -0.01 [-0.42, 0.38] | 0.948 |
| OME Opioids | 70 (84.3%) | -0.01 [-0.38, 0.40] | 0.965 |
| Phenylephrine | 66 (79.5%) | 0.26 [-0.08, 0.60] | 0.132 |
| Remifentanil | 6 (7.23%) | 0.19 [-0.35, 0.70] | 0.510 |
| aaMAC | 67 (80.7%) | 0.12 [-0.32, 0.51] | 0.632 |
| Propofol* | 83 (100%) | - | - |

*all patients received a non-zero dose of propofol.

**Supplemental Table 2.** Fisher’s z-transformed Spearman’s rho correlations [95% CI] between preoperative cognitive index values and intraoperative medication dosage.

| **Intraoperative Medications** | **Among the full patient cohort** | **P-Value** |
| --- | --- | --- |
| Acetaminophen (mg) | 0.01 [-0.21, 0.22] | 0.930 |
| Succinylcholine (mg) | 0.05 [-0.17, 0.26] | 0.677 |
| Ketorolac (mg) | 0.12 [-0.10, 0.33] | 0.286 |
| Cisatracurium (mg)^*^ | 0.10 [-0.12, 0.31] | 0.385 |
| Ketamine (mg) | -0.01 [-0.22, 0.21] | 0.954 |
| Midazolam (mg) | 0.13 [-0.09, 0.34] | 0.243 |
| Dexmedetomidine (µg) | 0.14 [-0.08, 0.35] | 0.196 |
| RE Paralytics (mg) | 0.10 [-0.12, 0.31] | 0.385 |
| OME Opioids (mg) | 0.15 [-0.07, 0.35] | 0.174 |
| Phenylephrine (µg) | -0.04 [-0.25, 0.18] | 0.714 |
| Remifentanil (µg) | 0.08 [-0.14, 0.29] | 0.498 |
| aaMAC Hours^**^ | -0.03 [-0.24, 0.19] | 0.804 |
| aaMAC | 0.05 [-0.17, 0.26] | 0.673 |
| Propofol (mg) | 0.11 [-0.11, 0.32] | 0.328 |

*only 1 patient received cisatracurium. **Among 66 patients with available aaMAC and surgical duration data.

**Supplemental Table 3.** Intraoperative parameters in the entire study cohort and in patients with vs. without postoperative delirium.

| **Intraoperative Parameters*** | **Overall N=83** | **No Postoperative Delirium N=71** | **Postoperative Delirium N=12** | **P-Value** |
| --- | --- | --- | --- | --- |
| Percent who received acetaminophen | 19 (22.89%) | 16 (22.54%) | 3 (25.00%) | 1.000^3^ |
| Acetaminophen (mg) | 1000 [1000, 1000] | 1000 [1000, 1000] | 1000 [1000, 1000] | 0.773^1^ |
| Percent who received succinylcholine | 12 (14.46%) | 11 (15.49%) | 1 (8.33%) | 1.000^3^ |
| Succinylcholine (mg) | 110 [100, 120] | 100 [100, 120] | 120 [120, 120] | 0.636^1^ |
| Percent who received ketorolac | 10 (12.05%) | 9 (12.68%) | 1 (8.33%) | 1.000^3^ |
| Ketorolac (mg) | 15 [15, 15] | 15 [15, 15] | 15 [15, 15] | 1.000^1^ |
| Percent who received cisatracurium** | 1 (1.20%) | 1 (1.41%) | 0 (0.00%) | 1.000^3^ |
| Percent who received ketamine | 24 (28.92%) | 21 (29.58%) | 3 (25.00%) | 1.000^3^ |
| Ketamine (mg) | 40 [30, 54.15] | 40 [35, 56.52] | 30 [20, 40] | 0.146^1^ |
| Percent who received midazolam | 14 (16.87%) | 13 (18.31%) | 1 (8.33%) | 0.681^3^ |
| Midazolam (mg) | 2 [2, 2] | 2 [2, 2] | 2 [2, 2] | 0.728^1^ |
| Percent who received dexmedetomidine | 18 (21.69%) | 16 (22.54%) | 2 (16.67%) | 1.000^3^ |
| Dexmedetomidine (µg) | 20 [8, 20] | 16 [8, 20] | 23.72 [20, 27.44] | 0.133^1^ |
| Percent who received paralytics | 68 (81.93%) | 56 (78.87%) | 12 (100.00%) | 0.112^3^ |
| RE^&^ Paralytics (mg) | 80 [65, 120] | 80 [60, 125] | 102.5 [75, 117.5] | 0.350^1^ |
| Percent who received opioids | 70 (84.34%) | 60 (84.51%) | 10 (83.33%) | 1.000^3^ |
| OME^&&^ Fentanil Hydromorphone (mg) | 20 [10, 25] | 15.89 [10.00, 25.00] | 21.25 [15.10, 27.67] | 0.359^1^ |
| Percent who received phenylephrine | 66 (79.52%) | 54 (76.06%) | 12 (100.00%) | 0.114^3^ |
| Phenylephrine (µg) | 2048 [300, 5051] | 2045 [300, 5590] | 2356 [894, 2777] | 0.555^1^ |
| Percent who received remifentanil** | 6 (7.23%) | 6 (8.45%) | 0 (0.00%) | 0.586^3^ |
| aaMAC Hours | 1.98 [1.29, 3.24] | 1.94 [1.27, 3.24] | 2.23 [1.91, 3.48] | 0.209^1^ |
| Mean end tidal aaMAC | 0.82 [0.7, 0.88] | 0.83 [0.69, 0.88] | 0.82 [0.76, 0.87] | 0.741^1^ |
| Propofol (mg) | 190 [120, 290] | 200 [120, 300] | 160.11 [130, 225] | 0.516^1^ |
| % of patients with BIS EEG | 57 (68.67%) | 51 (71.83%) | 6 (50.00%) | 0.179^3^ |
| Mean BIS* | 48.49 [45.6, 55.55] | 48.44 [45.60, 55.55] | 50.35 [40.56, 56.11] | 0.745^1^ |

Data are Mean (SD), Median [Q1, Q3], or N (%). ^1^Wilcoxon Rank Sums test, ^2^Chi-Square test, ^3^Fisher’s Exact test, ^4^T-test. *Among those who received the drug or who underwent BIS recording. **p-values could not be calculated for drug dosage (i.e., 0 patients in a group received the drug). aaMAC: age-adjusted minimum alveolar concentration. ^&^RE: rocuronium equivalent (RE = Rocuronium + (6/5)*Atracurium + 6*Vecuronium). ^&&^OME: oral morphine equivalent.
